# Combating acquired resistance to trastuzumab by an anti-ErbB2 fully human antibody

**DOI:** 10.18632/oncotarget.17451

**Published:** 2017-04-27

**Authors:** Chao Wang, Lingfei Wang, Xiaojie Yu, Yajun Zhang, Yanchun Meng, Huajing Wang, Yang Yang, Jie Gao, Huafeng Wei, Jian Zhao, Cuihua Lu, Han Chen, Yanping Sun, Bohua Li

**Affiliations:** ^1^ International Joint Cancer Institute and Department of Pharmaceutical Sciences, The Second Military Medical University, Shanghai, People's Republic of China; ^2^ Department of Medical Oncology, Fudan University Shanghai Cancer Center, Department of Oncology, Shanghai Medical College, Fudan University, Shanghai, People's Republic of China; ^3^ Shanghai Key Laboratory for Molecular Imaging, Shanghai University of Medicine and Health Sciences, Shanghai, People's Republic of China; ^4^ Department of Gastroenterology, The Affiliated Hospital of Nantong University, Nantong, People's Republic of China; ^5^ Department of General Surgery, 411 Hospital of Chinese People's Liberation Army, Shanghai, People's Republic of China; ^6^ Department of General Surgery, Changzheng Hospital, Second Military Medical University, Shanghai, People's Republic of China

**Keywords:** ErbB2, gastric cancer, acquired resistance to trastuzumab, domain I- specific antibody, programmed cell death

## Abstract

Trastuzumab resistance is a common problem that impedes the effectiveness of trastuzumab in ErbB2-amplified cancers. About 70% of ErbB2-amplified breast cancers do not respond to trastuzumab (de novo resistance), and the majority of the trastuzumab-responsive cancers progress within 1 year (acquired resistance). Different mechanisms exist between de novo and acquired resistance. Innate resistance mechanisms are mainly independent of ErbB2 receptor activity, and acquired resistance involves with alterations depending on ErbB2 activity. We previously reported H2-18, an ErbB2 domain I-specific antibody, which could circumvent de novo resistance to trastuzumab. Here, we modeled the development of acquired resistance by treating human gastric cancer cell line NCI-N87 with trastuzumab to obtain the trastuzumab-resistant subline, NCI-N87-TraRT. Next, we investigated the antitumor efficacy of H2-18 in NCI-N87-TraRT cell line. H2-18 exhibited a significantly greater antitumor activity in NCI-N87-TraRT tumor-bearing nude mice than pertuzumab and trastuzumab, either alone or in combination. The unique ability of H2-18 to overcome acquired resistance may be attributable to its potent programmed cell death-inducing activity, which was probably mediated by RIP1-ROS-JNK-c-Jun pathway. In conclusion, H2-18 may have the potential as an effective agent to circumvent acquired resistance to trastuzumab in ErbB2-overexpressing cancers.

## INTRODUCTION

Overexpression of ErbB2 is found in 25% to 30% of human breast cancers [1, 2] and about 4% to 50% of human gastric cancers according to different examination methods and criteria [3]. Trastuzumab, a therapeutic anti-ErbB2 humanized antibody, was approved by the US Food and Drug Administration (FDA) for clinical use for ErbB2-overexpressing metastatic breast cancer and metastatic gastric and gastro-esophageal junction cancer. Despite the effectiveness of trastuzumab, about 70% of patients could not respond to trastuzumab treatment (de novo resistance), and the majority of trastuzumab-responsive patients develop resistance within 1 year of treatment initiation (acquired resistance) [4–6]. The addition of pertuzumab, another anti-ErbB2 humanized antibody, to trastuzumab has enhanced the antitumor efficacy of trastuzumab and delayed trastuzumab resistance [7]. However, the objective response rate is only 24.2% and complete response only around 8% [8]. Thus, novel therapeutic anti-ErbB2 antibodies are urgently needed to overcome the resistance to trastuzumab-based therapy.

In the clinical treatment, the resistance to trastuzumab can be divided to two groups: intrinsic and acquired resistance. Intrinsic trastuzumab resistance is defined as lack of positive response to trastuzumab, whereas acquired resistance is clinically manifested as cancer progression after initial response to trastuzumab, or the stagnation of disease from treatment initiation over duration of six months [9, 10]. Many researches propose that the mechanisms underlying these two kinds of trastuzumab resistance are not totally overlapping. Therefore, the strategies which are effective in overcoming one resistance may be not a good choice for the other.

In our previous studies, H2-18, an ErbB2 domain I-specific fully human antibody, exhibited a greater antitumor effect than trastuzumab and pertuzumab, either alone or in combination, in innate trastuzumab resistant cancer cell lines [11, 12]. Here we investigated whether H2-18 could also overcome acquired resistance to trastuzumab in ErbB2-amplified cancers.

## RESULTS

### Establishment of trastuzumab-resistant gastric cancer cell line NCI-N87-TraRT

**Figure 1:**
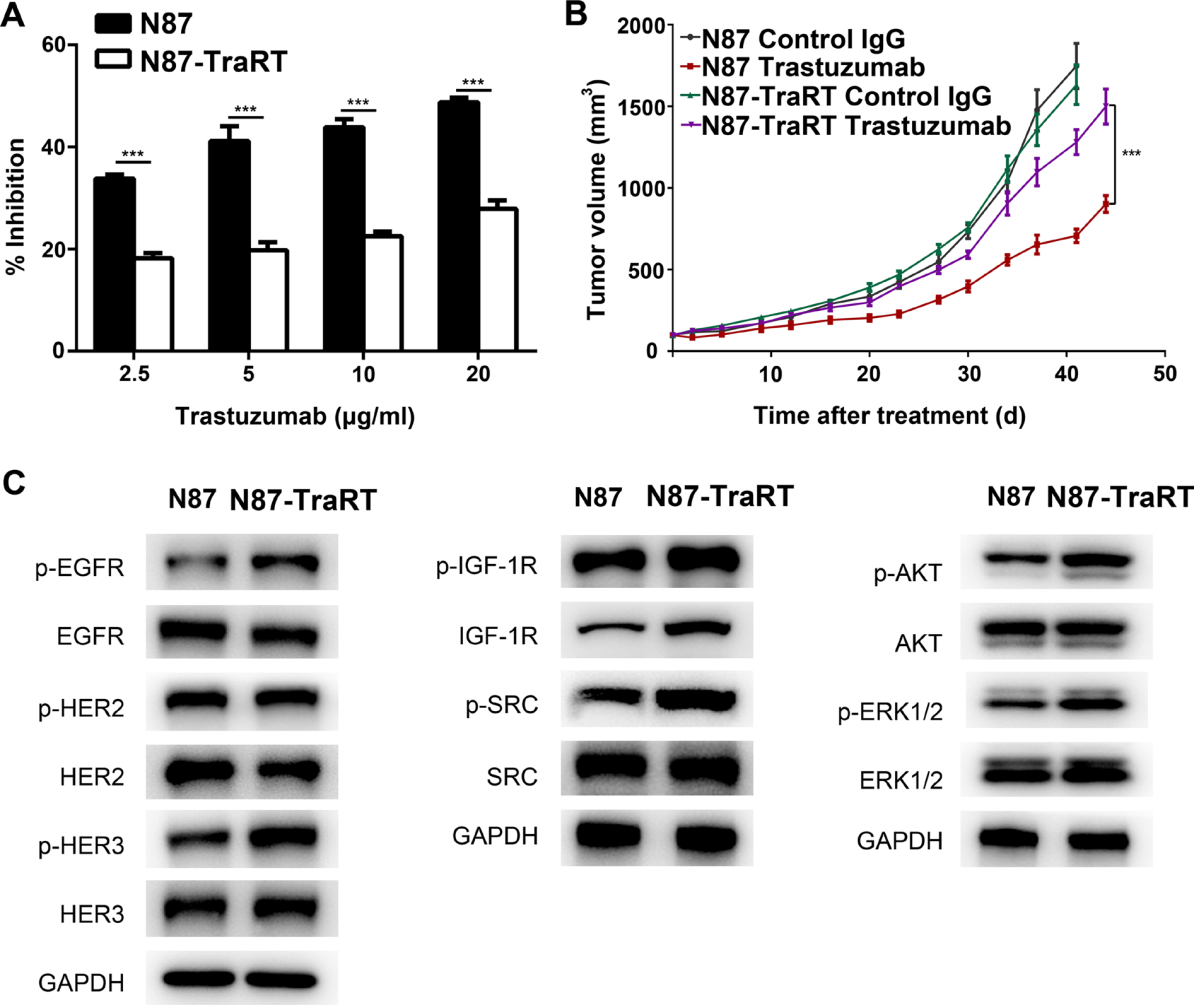
Establishment of trastuzumab-resistant gastric cancer cell line NCI-N87-TraRT (**A**), NCI-N87 and NCI-N87-TraRT cells were treated with an increasing concentration of trastuzumab for 5d. Cell proliferation was then determined by CCK8 assay. Results are shown as the inhibition rate of cell proliferation. Error bars, SD. ***P < 0.001; unpaired Student's *t-test*. (**B**), Tumor volume of NCI-N87 and NCI-N87-TraRT tumor xenografts after 4-week treatment with control IgG (10mg/kg, twice a week, intravenously) and trastuzumab (10 mg/kg, twice a week, intravenously). Data are shown as means ± SEM. ***P < 0.001, Mann-Whitney test. (**C**), Immunoblots comparing major cell signaling changes between NCI-N87 and NCI-N87-TraRT cell lines. Every experiment was repeated 3 times.

We modeled the development of acquired resistance in patients by treating human gastric cancer cell line NCI-N87 with trastuzumab to obtain the trastuzumab-resistant subline, NCI-N87-TraRT. Our data showed that NCI-N87-TraRT was more resistant to trastuzumab than NCI-N87 cell line, both *in vitro* and *in vivo* (Figure 1A, 1B). As tyrosine kinase receptors, which are ErbB2-like or partners of ErbB2, and some key molecules involving with ErbB2 signaling were reported to be upregulated in trastuzumab-resistant cancer cells [13–17], western blot was used to examine the level of EGFR, HER3, IGF1, AKT and ERK in H2-18-treated NCI-N87 and NCI-N87-TraRT cells. Compared with NCI-N87 cells, the phosphorylation of EGFR and ErbB3 were upregulated in NCI-N87-TraRT cells (Figure 1C). Additionally, IGF-1R, p-IGF-1R and p-SRC were also increased (Figure 1C). Moreover, p-Erk and p-Akt in NCI-N87-TraRT cell line were more than that in NCI-N87 cell line (Figure 1C). Thus, our results suggested that NCI-N87- TraRT cells were confirmed to acquire the resistance to trastuzumab.

### H2-18 exhibits *in vitro* anti-proliferation activity in both NCI-87 and NCI-N87-TraRT cell lines

**Figure 2:**
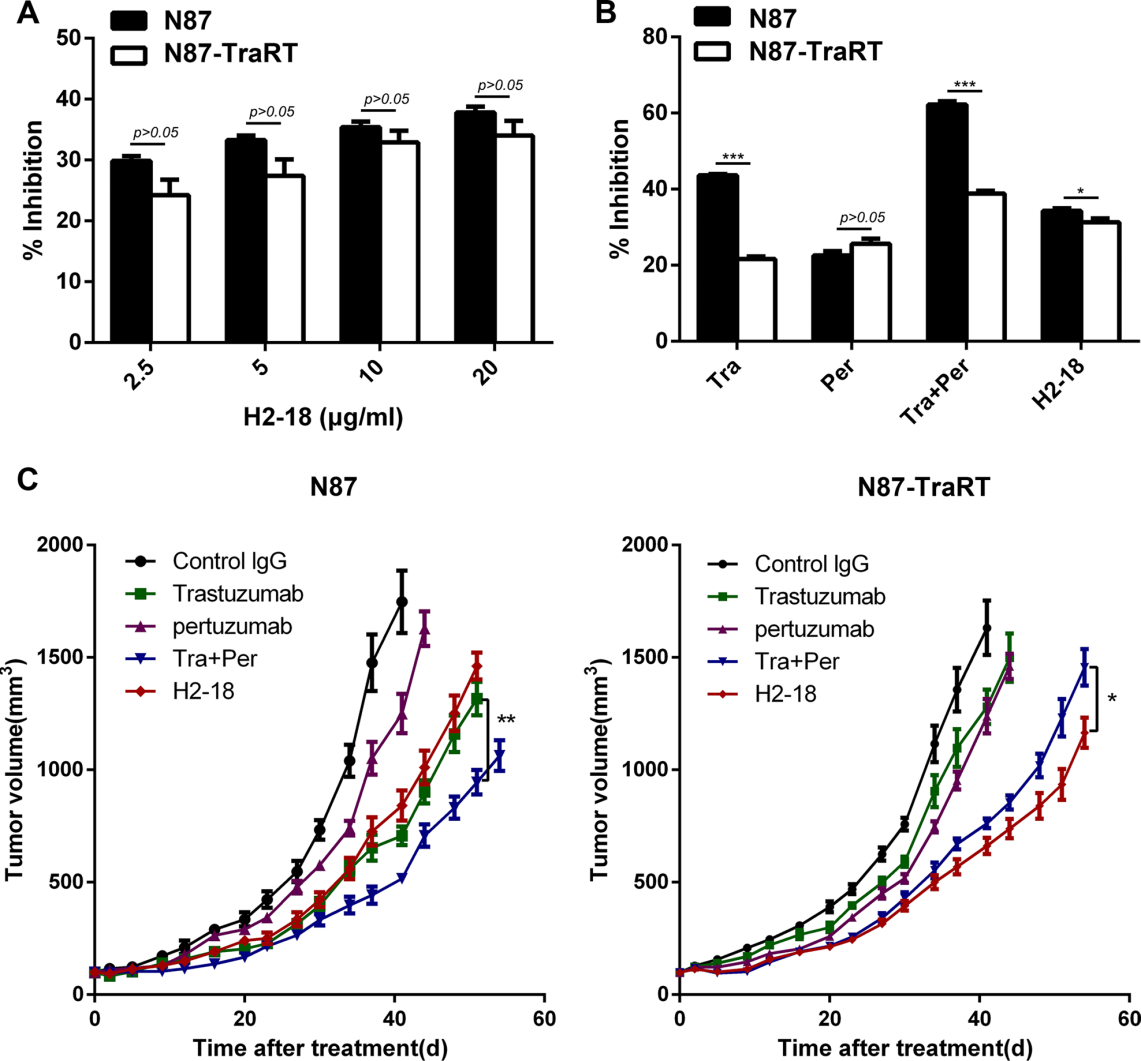
H2-18 effectively inhibits the growth of both NCI-87 and NCI-N87-TraRT tumors *in vitro* and *in vivo* (**A**), NCI-N87 and NCI-N87-TraRT cells were treated with an increasing concentration of H2-18 for 5d. Cell proliferation was then determined by CCK8 assay. Results are shown as the inhibition rate of cell proliferation. Error bars, SD. *P* > 0.05, unpaired Student's *t-test*. (**B**), NCI-N87 and NCI-N87-TraRT cells were treated with 10μg/ml of trastuzumab, pertuzumab, trastuzumab plus pertuzumab, and H2-18 for 5d. Cell proliferation was then determined by CCK8 assay. Results are shown as the inhibition rate of cell proliferation. Error bars, SD. *P < 0.05; **P < 0.01; ***P < 0.001; unpaired Student's *t-test*. (**C**), Tumor volume of NCI-N87 and NCI-N87-TraRT tumor xenografts after treatment with control IgG (10mg/kg, twice a week, intravenously), trastuzumab (10mg/kg, twice a week, intravenously), pertuzumab (10mg/kg, twice a week, intravenously), trastuzumab plus pertuzumab (5 mg/kg for each, twice a week, intravenously) and H2-18 (10 mg/kg, twice a week, intravenously). Data are shown as means ± SEM. *P < 0.05; **P < 0.01; Mann-Whitney test.

Next, we examined the anti-proliferation ability of H2-18 on NCI-87 and NCI-N87-TraRT cell lines. H2-18 could inhibit the proliferation of both NCI-N87 and NCI-N87-TraRT cell lines in a dose-dependent manner (Figure 2A). In the trastuzumab-sensitive gastric cancer cell line NCI-N87, although H2-18 was more effective in inhibition of cell growth than pertuzumab, the cell proliferation inhibition effect of H2-18 was weaker than that of either trastuzumab or trastuzumab plus pertuzumab (Figure 2B). However, in the trastuzumab-resistant gastric cancer NCI-N87-TraRT cell line, H2-18 showed a greater ability to inhibit cell proliferation compared with trastuzumab and pertuzumab alone (Figure 2B).

### H2-18 effectively inhibits the *in vivo* growth of both NCI-87 and NCI-N87-TraRT tumors

The *in vivo* antitumor efficacy of trastuzumab, pertuzumab, trastuzumab plus pertuzumab and H2-18 were examined in nude mice bearing trastuzumab-sensitive NCI-N87 or trastuzumab-resistant NCI-N87-TraRT tumor xenografts. Trastuzumab was more effective than pertuzumab in inhibiting NCI-N87 tumor, whereas trastuzumab plus pertuzumab exhibited a greater inhibitory ability than either antibody alone (Figure 2C). And the *in vivo* inhibitory effect of H2-18 on NCI-N87 tumor was similar to that of trastuzumab (Figure 2C). Importantly, H2-18 could inhibit the *in vivo* growth of NCI-N87-TraRT tumor more effectively than trastuzumab and pertuzumab, either alone or in combination (Figure 2C). Thus, H2-18 may be a more potent antitumor drug than trastuzumab plus pertuaumzb in ErbB2-amplified tumor that was acquired resistant to trastuzumab.

### H2-18 inhibits the downstream signaling pathways of ErbB2 in NCI-N87 cells but not in NCI-N87-TraRT cells

**Figure 3:**
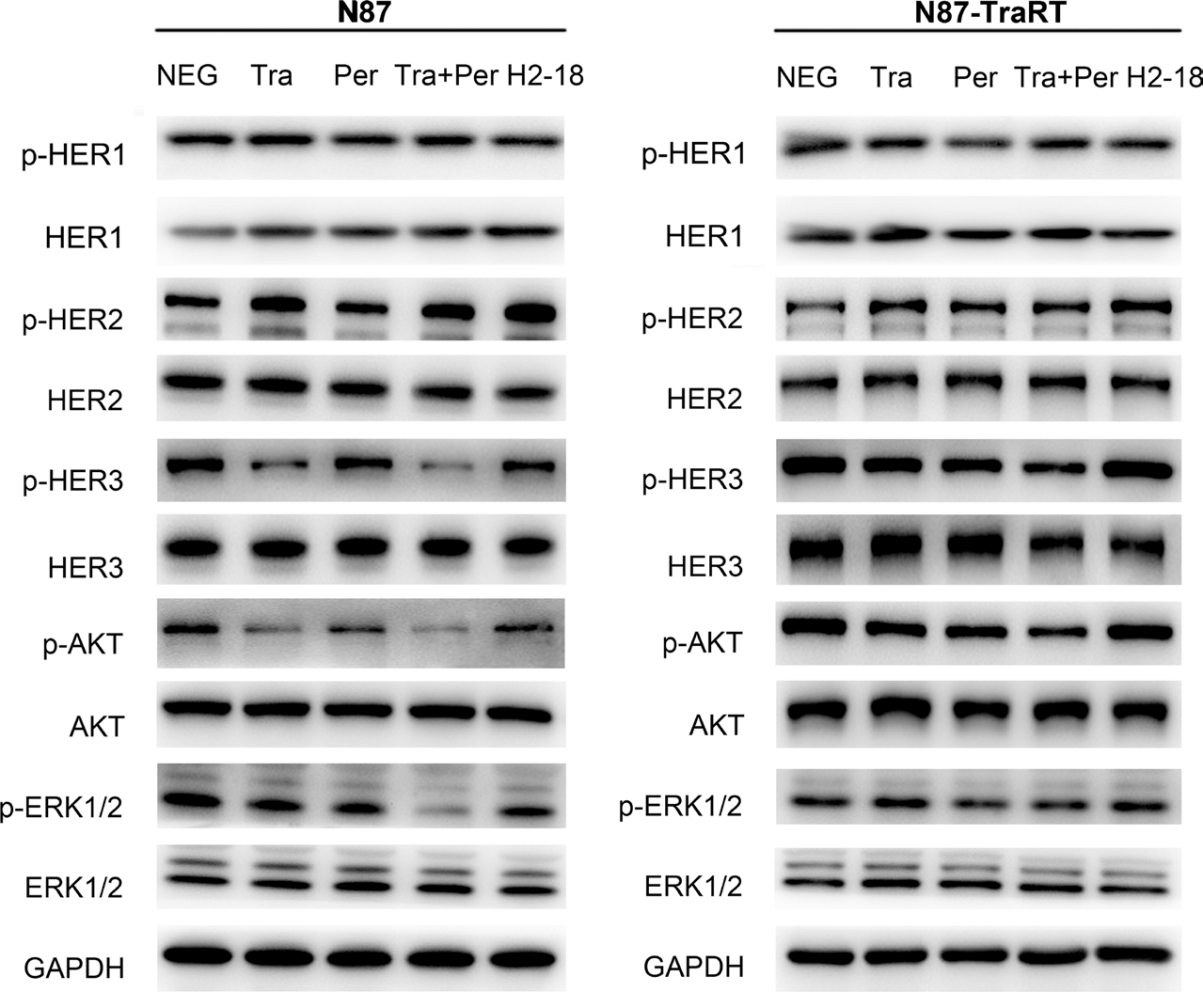
H2-18 inhibits the downstream signaling pathways of ErbB2 in NCI-N87 cells but not in NCI-N87-TraRT cells Immunoblots assessing the effect of control IgG (NEG), trastuzumab, pertuzumab, trastuzumab plus pertuzumab, and H2-18 on ErbB2 signaling changes in NCI-N87 and NCI-N87-TraRT cell lines. Every experiment was repeated 3 times.

Western blot was used to determine the changes of ErbB2 signaling in NCI-N87 cells and NCI-N87-TraRT cells treated with control IgG, trastuzumab, pertuzumab, trastuzumab plus pertuzumab, and H2-18. In trastuzumab-sensitive gastric cell line NCI-N87, trastuzumab could effectively inhibit p-Akt and p-Erk, the key downstream signaling molecules of ErbB2 (Figure 3). On the contrary, the inhibitory effect of trastuzumab on phosphorylation of both Akt and Erk was greatly weakened in trastuzumab-resistant NCI-N87-TraRT cells (Figure 3). Compared with trastuzumab or pertuzumab alone, trastuzumab plus pertuzumab exhibited the most potent ability to decrease p-HER3, p-Akt and p-Erk in NCI-N87 and NCI-N87-TraRT cells (Figure 3). H2-18 could reduce the phosphorylation of HER3, Akt and Erk in NCI-N87 cells, but the inhibitory effect was less strong than trastuzumab plus pertuzumab. In H2-18-treated NCI-N87-TraRT cells, no significant reduction of p-HER3, p-Akt and p-Erk were observed (Figure 3). In both NCI-N87 and NCI-N87-TraRT cell lines, no significant difference was obtained with HER1 and p-HER1 among the indicated groups (Figure 3). Therefore, our results indicated that inhibition of classical ErbB2 signaling pathways was not sufficient to account for the *in vitro* and *in vivo* antitumor effect of H2-18.

### H2-18 potently induces cell death in both NCI-87 and NCI-N87-TraRT cell lines

**Figure 4:**
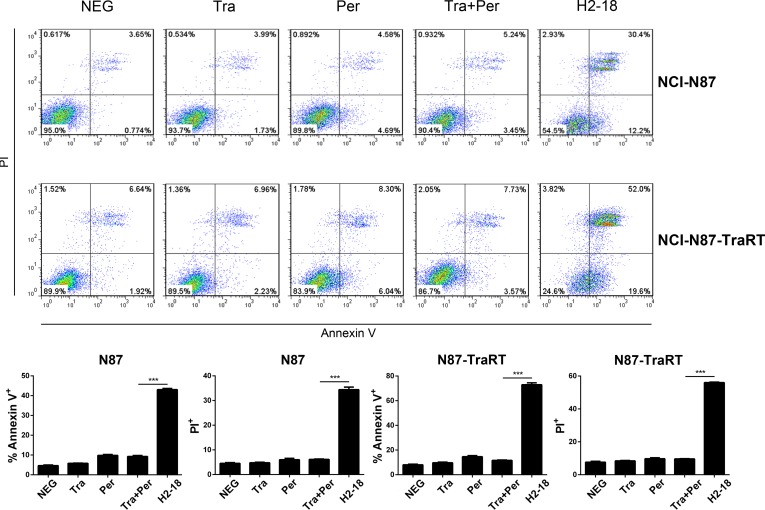
H2-18 potently induces cell death in both NCI-87 and NCI-N87-TraRT cell lines Cell death induced by control IgG, trastuzumab, pertuzumab, trastuzumab plus pertuzumab, and H2-18 in NCI-N87 and NCI-N87-TraRT cell lines was measured by flow cytometry using Annexin V/PI detecting kit. Every experiment was repeated 3 times. Error bars, SD. ***P < 0.001; unpaired Student's *t-test*.

**Figure 5:**
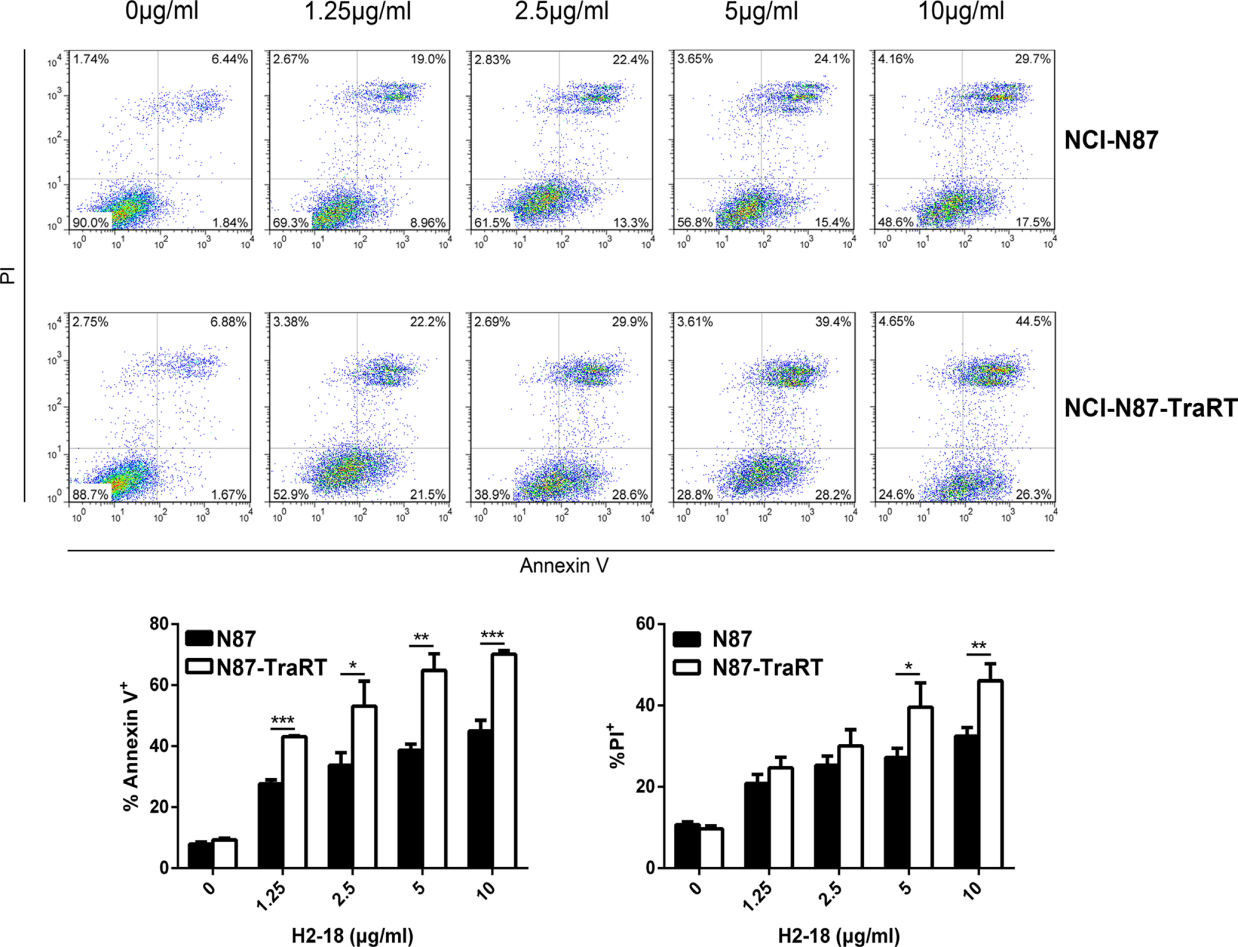
H2-18 induces cell death in a dose-dependent manner Cell death induced by an increasing concentration of H2-18 in NCI-N87 and NCI-N87-TraRT cell lines was determined by flow cytometry using Annexin V/PI detecting kit. Every experiment was repeated 3 times. Error bars, SD. *P < 0.05; **P < 0.01; ***P < 0.001; unpaired Student's *t-test*.

Flow cytometry was performed to compare the cell death-inducing ability of trastuzumab, pertuzumab, trastuzumab plus pertuzumab, and H2-18 in NCI-N87 and NCI-N87-TraRT cells. The percentage of Annexin V-positive H2-18-treated NCI-N87-TraRT cells was 71.6%, far higher than that of NCI-N87-TraRT cells treated with trastuzumab and pertuzumab, either alone or in combination (around 10%) (Figure 4). H2-18 could also induce much more PI-positive NCI-N87-TraRT cells than did all the other indicated anti-ErbB2 antibodies (Figure 4). Similar results were obtained inNCI-N87 cells (Figure 4). The results showed that H2-18 could induce cell death more potently than the other mAbs, or the mAb combination. In addition, H2-18 induced cell death in a dose-dependent manner in both gastric cancer cell lines (Figure 5). Interestingly, H2-18 exerted a more potent cell death-inducing ability in NCI-N87-TraRT cells than that in NCI-N87 cells (Figure 5). Thus, H2-18 exhibited a potent cell death-inducing ability especially in trastuzumab-resistant cell line NCI-N87-TraRT.

### H2-18 increases ROS in both NCI-N87 and NCI-N87-TraRT cell lines

**Figure 6:**
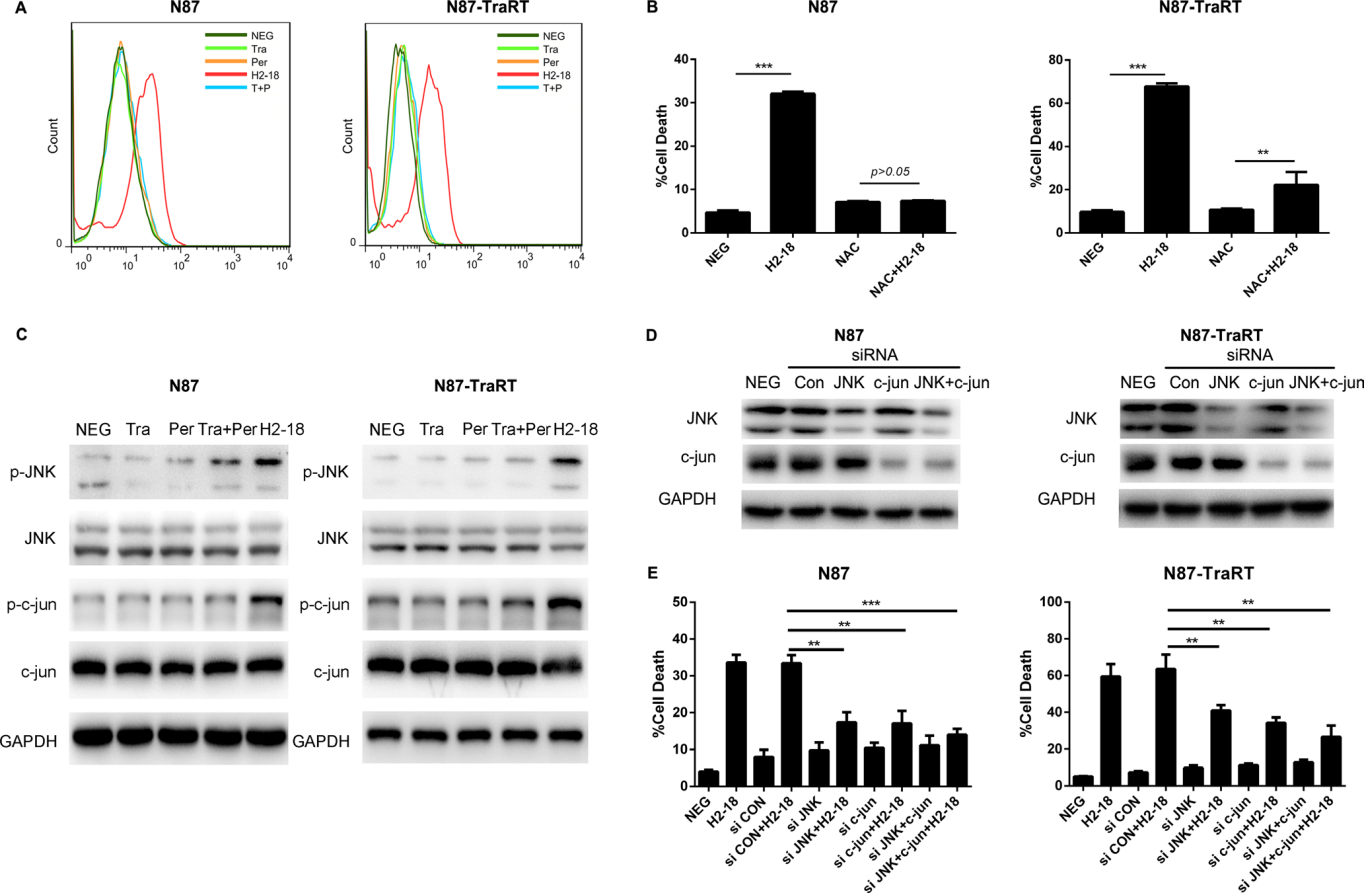
H2-18 increases ROS and p-JNK/p-c-Jun in both NCI-N87 and NCI-N87-TraRT cell lines (**A**), Flow cytometry was used to examine the ROS production in NCI-N87 and NCI-N87-TraRT cells treated with control IgG, trastuzumab, pertuzumab, pertuzumab plus trastuzumab, and H2-18. (**B**), The effect of ROS scavenger NAC on H2-18-induced cell death in NCI-N87 and NCI-N87-TraRT cells. Data are shown as means ± SD. **P < 0.01; ***P < 0.001; unpaired Student's *t-test*. (**C**), Immunoblots comparing the effect of control IgG (NEG), trastuzumab, pertuzumab, trastuzumab plus pertuzumab, and H2-18 on JNK and c-Jun activation in NCI-N87 and NCI-N87-TraRT cell lines. Every experiment was repeated 3 times. (**D**), Immunoblots confirming the gene knockdown effect of siRNA in NCI-N87 and NCI-N87-TraRT cell lines. (**E**), The effect of knockdown of JNK, c-Jun, and JNK plus c-Jun on H2-18-induced cell death in NCI-N87 and NCI-N87-TraRT cells. Data are shown as means ± SD. **P < 0.01; ***P < 0.001; ANOVA.

Trastuzumab and pertuzumab, either alone or in combination, did not increase ROS production in both NCI-N87 and NCI-N87-TraRT cell lines. However, the ROS level was significantly induced by H2-18 (Figure 6A). Next, we determined the cell death induced by H2-18 with or without the ROS scavenger N-acetyl-L-cysteine (NAC). Our results showed that NAC could nearly abrogate the cell death-inducing ability of H2-18 in both NCI-N87 and NCI-N87-TraRT cell lines (Figure 6B). Thus, increased ROS production induced by H2-18 results in programmed cell death.

### H2-18 upregulates p-JNK and p-c-Jun in both NCI-N87 and NCI-N87-TraRT cell lines

Western blot was used to assess the effect of trastuzumab, pertuzumab, trastuzumab plus pertuzumab, and H2-18 on JNK/c-Jun activation. Trastuzumab and pertuzumab, either alone or in combination, have no effect on phosphorylation of both JNK and c-Jun, whereas H2-18 significantly increased p-JNK and p-c-Jun in NCI-N87 and NCI-N87-TraRT cell lines (Figure 6C). Next, we knocked down the expression of JNK, c-Jun, or both of them using specific small-interfering RNAs (siRNAs). In both NCI-N87 and NCI-N87-TraRT cell lines, all siRNA knockdown of JNK, c-Jun, or both of them could partially abolished H2-18-induced cell death (Figure 6D, 6E). Collectively, H2-18 treatment leads to JNK/c-Jun activation, which is reported to contribute to induction of cell death.

## DISCUSSION

The potential mechanisms underlying trastuzumab resistance have been widely studied, including mechanisms dependent on ErbB2 receptor activity and those independent of ErbB2 receptor activity [13, 14]. Mechanisms that are dependent on ErbB2 receptor activity includes overexpression of proteins that masks ErbB2 receptor, upregulation of target-like tyrosine kinase receptors or their ligands, and formation of truncated ErbB2 [13, 14]. In contrast, mechanisms independent of ErbB2 receptor activity covers alterations of downstream components in PI3K/Akt signaling pathway, cell reprogramming by deregulation of anti-apoptotic proteins or cell cycle regulators, and EMT transition [13, 14]. Difference indeed exists between instinct and acquired resistance mechanisms [13]. Up to 70% of intrinsic resistance mechanisms do not overlap with acquired resistance mechanisms [18].

The dominant mechanisms of intrinsic resistance are independent of ErbB2 receptor activity, relating to inactive ErbB2 receptor and aberrations located in downstream signals [13]. The predominant resistance-generating alterations were PI3KCA mutation and loss of PTEN, which lead to persistent activation of PI3K/AKT signaling pathway [14, 18–21]. In our previous study, HCC-1954 harbors an activating PIK3CA mutation (H1047R). Both HCC-1954 and HCC-1419 have low expression of PTEN [20]. H2-18, an ErbB2 domain I-specific antibody, showed the ability to overcome trastuzumab resistance in intrinsic resistant cell lines especially HCC-1954 [12].

Different from de novo resistance, almost all the acquired trastuzumab resistance mechanisms depend on ErbB2 receptor activity, involving active ErbB2 and ErbB2-dependent aberrations located on the target signal level [13]. Taking into account that intrinsic resistance exists before trastuzumab application, it is viable to produce acquired resistant model by selective pressure of trastuzumab on trastuzumab-sensitive cells. Here, we developed an ErbB2-amplied acquired resistant model NCI-N87-TraRT from the trastuzumab-sensitive cell line NCI-N87. Compared with NCI-N87, the co-receptors of ErbB2 (EGFR and ErbB3) were activated in NCI-N87-TraRT. IGF-1R and p-IGF-1R were also upregulated. In addition, the phosphorylation of downstream molecules PI3K, MAPK and SRC were also observed in NCI-N87-TraRT. Our results indicated that upregulated activity of RTK, with HER2 as a preferential dimerization receptor partner, may contribute to acquired resistance to trastuzumab. Although H2-18 did not inhibit *in vitro* cell proliferation of NCI-N87 and NCI-N87-TraRT cells as effectively as trastuzumab plus pertuzumab, H2-18 was more effective than trastuzumab plus pertuzumab in inhibiting the *in vivo* growth of NCI-N87-TraRT tumors. Further studies showed that H2-18 could induce cell death far more potently than trastuzumab plus pertuzumab. As is suggested in our previous study with de novo resistant models, the enhanced programmed cell death (PCD)-inducing ability of the H2-18 antibody was responsible for its antitumor efficacy [12]. And PCD induced by H2-18 was mainly mediated by RIP1-ROS-JNK-c-Jun signaling pathway [12]. Here, p-JNK and p-c-Jun were increased in both NCI-N87 and NCI-N87-TraRT cell lines. The level of ROS was also significantly induced by H2-18. Furthermore, the ROS scavenger NAC was able to abrogate the cell death-inducing ability of H2-18. Thus, we concluded that the superior *in vivo* antitumor efficacy of H2-18 in NCI-N87-TraRT cells may be also attributable to its potent PCD-inducing activity, dependent on RIP1-ROS-JNK-c-Jun pathway.

The mechanisms underlying trastuzumab resistance are quite complicated. They vary in clinical cases, which exert ErbB2 overexpression but ineffectively respond to trastuzumab. Instinct and acquired resistance, the clinical types of trastuzumab resistance, were developed mainly through different alterations. Our study showed that H2-18 could circumvent the both de novo and acquired resistance to trastuzumab, suggesting that the induction of programmed cell death may be an effective strategy to overcome trastuzumab resistance. In conclusion, H2-18 may have the potential to circumvent trastuzumab resistance in ErbB2-amplified cancers.

## MATERIALS AND METHODS

### Antibodies, cell lines, and animals

H2-18 was expressed and purified using the method as described in our previous report [11]. Trastuzumab was purchased from Roche Ltd. The pertuzumab antibody [22] was expressed and purified using the method described in our previous studies [23]. The ErbB2-overexpressing human gastric cancer cells NCI-N87 were purchased from the American Type Culture Collection (ATCC). Trastuzumab-resistant subline NCI-N87-TraRT cell line was developed from NCI-N87 cell line, which was treated with 10μg/ml trastuzumab consecutively for 2 years. All the cells were authenticated twice by morphologic and isoenzyme analyses during the study period. Cell lines were routinely checked for contamination by mycoplasma using Hoechst staining and consistently found to be negative. Six-week-old female BALB/c nude mice were obtained from the Shanghai Experimental Animal Center of Chinese Academy of Sciences. All animals were treated in accordance with guidelines of the Committee on Animals of the Second Military Medical University.

### Cell proliferation assay

Cells were seeded at a density of 3× 10^3^ cells per well in a 96-well plate in a humidified 37°C and 5% CO_2_ atmosphere. After a 24-h attachment, the cells were treated with 10 μg/ml of control IgG, trastuzumab, pertuzumab, trastuzumab plus pertuzumab, and H2-18 for an additional 5 days. Medium was refreshed every 2 or 3 days. Cell proliferation was determined by Cell Counting Kit CCK-8/WST-8 (DOJINDO, Japan).

### Immunoblotting

Cells were treated with control IgG (10 μg/ml), trastuzumab (10 μg/ml), pertuzumab (10 μg/ml), trastuzumab plus pertuzumab (10 μg/ml for each), and H2-18 (10 μg/ml) for 4 h at 37°C. After washing twice with PBS, cells were lysed in SDS lysis buffer and then were subjected to SDS-PAGE. The proteins were transferred to polyvinylidene difluoride (PDVF) membrane and immunoblotted with indicated primary antibodies and Horseradish peroxidase (HRP)-conjugated secondary antibodies (all from Cell Signaling Technology). Finally, detection was performed using the enhanced chemiluminescence reagents (Plus-ECL, PerkinElmer, MA, USA) or chemiluminescence reagents (Millipore, MA, USA).

### Cell death assay

Cells were seeded in flat-bottomed 24-well plate at a density of 1 × 10^5^ cells per well in the growth medium and grown overnight at 37°C in a humidified incubator with 5% CO_2_. Next day, cells were treated with 10 μg/ml of control IgG, trastuzumab, pertuzumab, trastuzumab plus pertuzumab, and H2-18 for 24 h. Cell death was measured by using Dead Cell Apoptosis Kit with Annexin V Alexa Fluor^®^ 488 & Propidium Iodide (PI) (Life Technologies) according to the manufacturer's protocol. Briefly, cells were harvested, washed twice with binding buffer and then resuspended in 100 μl binding buffer per tube. Five microliter 488-conjugated Annexin-V and 0.1 μl PI were added to cell suspension. After 15 min incubation at room temperature, the cells were washed with binding buffer and resuspended in 200 μl binding buffer, then analyzed by flow cytometry on a FACSCalibur (Becton Dickinson).

### Inhibitor assay

For the inhibitor assay, overnight grown NCI-N87 or NCI-N87-TraRT cells were first treated with 10 mM NAC (a ROS scavenger, selleck) for 2 h before H2-18 was cultured with the cells. After 24 h incubation at 37°C, cell death assay was carried out as described in previous section.

### Detection of ROS production

The ROS level in NCI-N87 and NCI-N87-TraRT cells was evaluated with total reactive oxygen species (ROS) assay kit (eBioscience). NCI-N87 and NCI-N87-TraRT cells were seeded in a flat-bottomed 24-well plate. Next day, cells were treated with indicated anti-ErbB2 mAbs or the mAb combination for indicated time. Then, cells were harvested and incubated with ROS assay stain for 60 min at 37°C. Then, the fluorescence of the cells was measured with FACSCalibur (Becton Dickinson) using excitation wavelength of 488 nm and emission wavelength of 520 nm.

### Transfection of small interfering RNA (siRNA)

Transfection of siRNA was conducted using DharmaFECT 4 transfection reagent (Dharmacon) according to the manufacturer's instructions. The control siRNA (5′-UUC UCC GAA CGU GUC ACG UTT-3′), JNK siRNA (5′- GCC CAG UAA UAU AGU AGU ATT-3′), and c-Jun siRNA(5′-ACG CAA ACC UCA GCA ACU UTT-3′) were purchased from GeneParma Company (Suzhou, China). NCI-N87 and NCI-N87-TraRT cells were transfected with siRNA for 48 hour and then subjected to western blot. After siRNA transfection for 48 hour, the cells were incubated with or without H2-18 for another 24 h before cell death assay.

### *In vivo* therapy study

Six-year-old female BALB/c nude mice were implanted with NCI-N87 or NCI-N87-TraRT cells (1 × 10^7^ per mouse) in right mammary fat pad. When tumors reached an average volume of 100 mm^3^, mice were grouped to treatment cohorts of 10 mice each randomly. Control IgG (10 mg/kg), trastuzumab (10 mg/kg), pertuzumab (10 mg/kg), trastuzumab plus pertuzumab (5 mg/kg for each), and H2-18 (10 mg/kg) were injected intravenously twice a week for 4 weeks. Tumors were measured with digital calipers twice a week and tumor volumes were calculated by the formula: volume (mm^3^) = length × (width) ^2^ /2.

### Statistical analysis

The data were tested for parametric distribution before applying parametric analysis. Statistical analysis was conducted by unpaired Student's *t-test* to identify significant differences unless otherwise indicated. Differences were considered significant at *P* < 0.05.
